# Expanding the reach of global health radiology via the world’s first medical hybrid airship: A SWOT analysis

**DOI:** 10.7189/jogh.10.010374

**Published:** 2020-06

**Authors:** Rameiya Paramalingam, Ryan England, Daniel Mollura, David Koff

**Affiliations:** 1 Faculty of Health Sciences, McMaster University, Hamilton, Ontario, Canada; 2 Department of Radiology, Johns Hopkins Hospital, Baltimore, Maryland, USA; 3 RAD-AID International, Chevy Chase, Maryland, USA; 4 Department of Radiology, McMaster University, Hamilton, Ontario, Canada

Radiology is a specialty in medicine that uses various medical imaging (MI) modalities, such as x-ray radiography, computed tomography (CT), ultrasound, and magnetic resonance imaging (MRI), to produce and interpret images for prevention, diagnosis and treatment of disease [1]. MI is a core health care technology, listed by the World Health Organization (WHO) as one of the six essential building blocks for health systems to operate smoothly [2]. Additionally, access to radiology services can influence six of the United Nations’ Sustainable Development Goals (SDGs): maternal health (3.1), child health (3.2), communicable diseases such as tuberculosis (TB) and human immunodeficiency virus (HIV) (3.3), non-communicable diseases such as cancer (3.4), road traffic accidents (3.6), and universal health coverage (3.8) [3]. However, three to four billion individuals worldwide lack access to basic radiology services and other important health care services [4]. The disparity in access exists within high-income countries and between high-income countries and low to middle income countries (LMICs). Thus, prioritizing global radiology is fundamental in promoting the SDGs agenda and improving health care outcomes.

## MOBILE HEALTH IN RADIOLOGY

Mobile health care programs have been developed to target the lack of access to radiology services worldwide [5,6]. The goal of mobile health is to bring health care to populations through different modes of transportation such as trucks, ships, and aircraft, to overcome geographic, structural and sociocultural barriers. Geographic barriers include physical isolation and far travel distance to health clinics. Structural barriers include absent or inadequate transportation infrastructure and long wait times. Sociocultural barriers include low socioeconomic status, education level and social norms.

Mobile health units (MHUs) are a form of mobile health care that delivers health care services throughout a community using a road unit such as a van or truck. The use of MHUs dates back to World War I, when imaging techniques were provided to soldiers by surgeons during battle in remote areas [7]. The cost-effective services, compact nature, and social acceptability are notable benefits of the MHUs [8]. Yet, as much of MI technology consists of large, heavy, and delicate equipment, inadequate road infrastructure prohibits successful implementation of MHUs in many parts of the world. Moreover, the World Bank’s Rural Access Index estimates that approximately 1 billion people live without access to reliable infrastructure, defined as living within 2 km of an all-season traversable road [8]. The use of traditional modes of transportation as a mechanism to deliver radiology services must therefore be reevaluated.

## THE MEDICAL HYBRID AIRSHIP

RAD-AID International, a non-profit organization that focuses on improving access to basic radiology services and technology worldwide, along with Lockheed Martin and Straightline Aviation, collaborated to develop the world’s first Medical Hybrid Airship (MHA). The MHA program has been developed to overcome transportation and geographic barriers that impede individuals from accessing radiology services [4]. The airship is capable of carrying and delivering advanced MI technology via deployable mobile health units with up to 18 personnel to the most remote and resource limited regions of the world.

**Figure 1:**
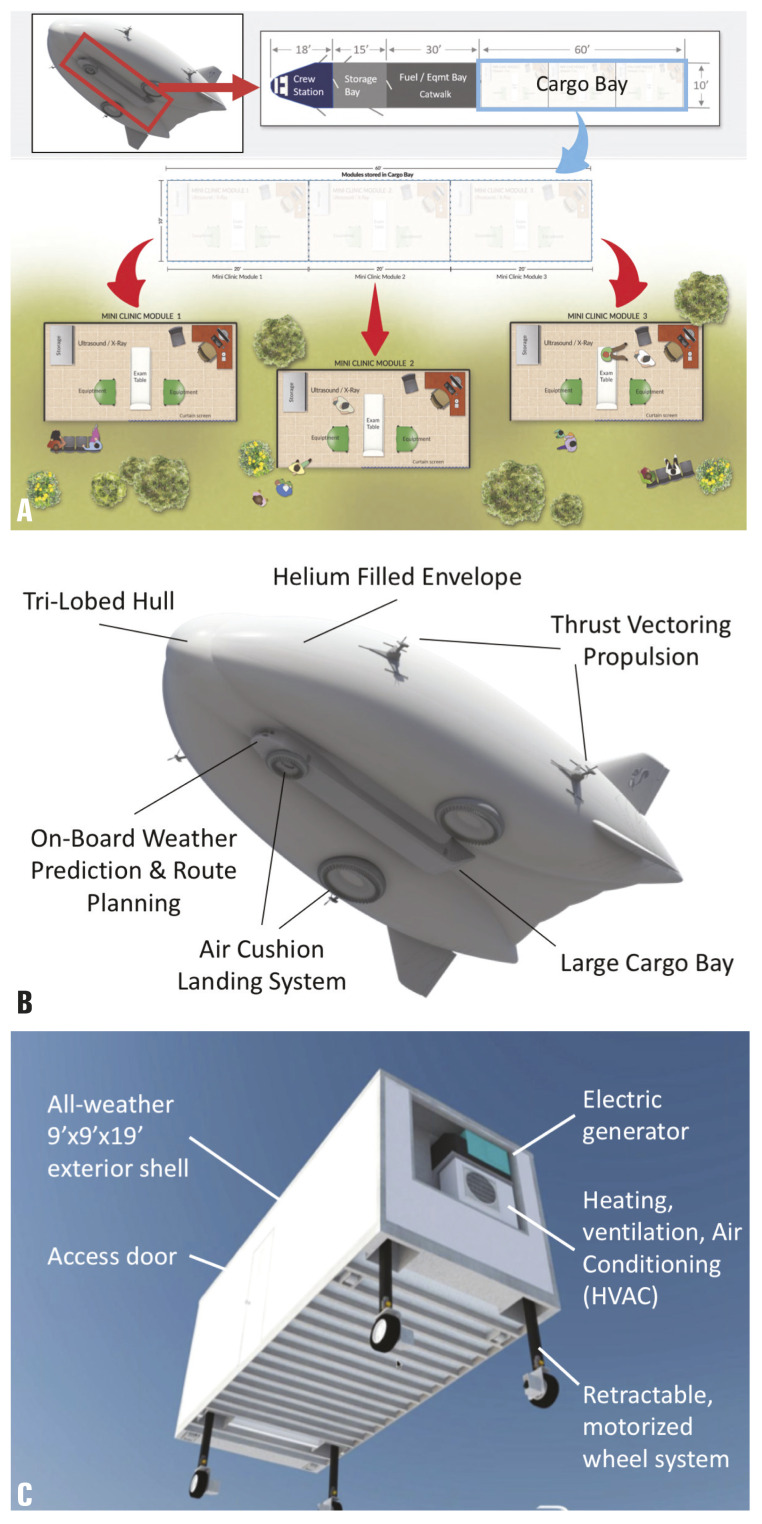
The RAD-AID medical hybrid airship. **Panel A.** The RAD-AID medical hybrid airship cargo area and mobile health unit designs. **Panel B.** The air cushion landing system (ACLS) of the RAD-AID medical hybrid airship. **Panel C.** Engineered design of the mobile health unit and deployable wheel system. Credit: RAD-AID International, with permission.

This new approach to global radiology outreach utilizes both buoyancy and propulsion to carry up to 20 tons of heavy and delicate radiology technology over long distances. Its ability to land and takeoff on any flat unprepared surface (eg, grass, sand, snow, water, etc.) utilizing an air cushion landing system (ACLS) allows the airship to reach the most rural regions (**Figure 1**). Capable of operating in temperatures from -40 to 120°F (-40 to 49°C), the airship can perform operations in various climates ranging from the arctic tundra of northern Canada to the dry heat of Africa. The estimated number of operational flying days of the airship is 300 days annually and requires 1/10^th^ of the fuel compared to traditional heavy-lift helicopters [4].

Furthermore, the cargo space within the airship spans 60’ × 10’ × 10’ (18.2 m × 3 m × 3 m) which allows mini-clinics to be loaded in and deployed from the airship. The deployable mini-clinics are designed to be approximately 9’ × 9’ × 18’ (2.7 m × 2.7 m × 5.5 m) and can be tailored to address location specific needs to increase the efficiency of the service in that region (**Figure 1**). In addition to containing radiology tools and capabilities, deployable clinics are designed with electric generators and environmental comfort via heating, ventilation, and air conditioning (HVAC) systems.

## SWOT ANALYSIS OF THE AIRSHIP PROGRAM

In the development and deployment of new and evolving technology, SWOT (Strengths, Weakness, Opportunity, Threats) analyses can be a helpful construct for identifying challenges and producing solutions. As radiology is a quickly evolving technological segment of the health care sector, and hybrid airship technology is relatively new to the aircraft and air transportation sectors, a SWOT analysis provides a useful means for evaluating the next steps in planning mobile radiology and long-term humanitarian aid transport.

### Strengths

As MI technology can be heavy and delicate, the MHA is uniquely capable of transporting sensitive materials to previously inaccessible areas through a near-vibration-free mode of flight. The high cargo tonnage capability of the MHA also allows for the moving of heavy equipment over long distances of over 1500 miles. The MHA program promotes operational sustainability by establishing bases of operation for the resupply of equipment and transfer of personnel. The appropriateness of provided radiology services to outreach areas will be ensured through the use of the RAD-AID Radiology-Readiness Assessment, to assess the existing radiology structure and health care needs of a specific region prior to designing and planning an outreach project [9]. The MHA program will also use geographic information systems (GIS), which manage and interpret geographical data for analysis, as a planning tool to determine potential landing zones for the airship [4]. Moreover, the MHA has a lower environmental impact and cost than other flight and road transportation networks, offering ecofriendly and efficient ways of delivering advanced health care.

**Figure:**
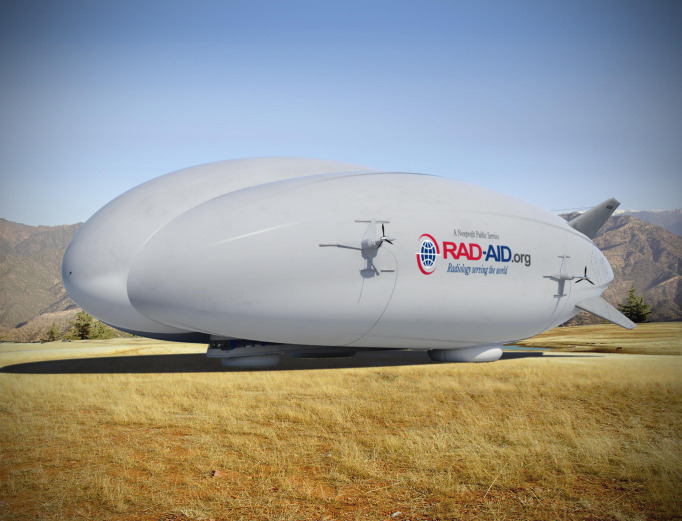
Photo: The RAD-AID medical hybrid airship (Credit: RAD-AID International, used with permission).

### Weaknesses

Although GIS is capable of creating maps of clinical data superimposed on topographical data of a region for navigational planning, GIS databases currently lack access to local clinical outcomes. This information is important for pre-planning as it can provide baseline data against which the program can make adjustments to address population specific gaps in health care. Another challenge in mobile health care is a compromised patient follow-up system due to a weak or absent referral network. A referral network allows for continuing patient care through MHUs integrating with larger health care facilities in the local environment. In comparison to traditional health care facilities, mini-clinic or MHUs are much smaller and may limit the presence of all stakeholders in a radiology organization (eg, medical radiation technologists, nurses, radiologists, and medical physicists). Additionally, consistent staffing can be compromised due to the nature of the small workspace and risks associated with working in an underserved and remote community. This can subsequently lead to scheduling constraints. A final weakness is how operational costs are distributed. Although the overall cost of airship-based mobile health for remote regions is estimated to be lower in terms of total infrastructure and environmental costs than conventional flight and road-based transport, the absence of infrastructure paid for by public sources as a collective good means that the costs are less broadly distributed. Total costs are therefore more focused on the MHA program operators, giving the appearance of higher costs. These differences in cost-distribution are important when aligning stakeholders and calculating relative costs and investment.

### Opportunities

The MHA program can facilitate the involvement and engagement of local community members through providing formalized training to lay health workers (LHWs) in local health care facilities or clinics [10]. By empowering local community members and LHWs to become important and contributing members of their community’s health care system, the quality, accessibility and sustainability of services should improve significantly [10]. The MHA program can offer opportunities in health systems research to promote advancements in the MHA program and adjustments in strategic approaches. In addition, the MHA program can serve as an opportunity to integrate with local health facilities and organizations to strengthen referral networks and disseminate knowledge to increase public awareness of the MHA program.

### Threats

The MHA program can be threatened due to extreme weather conditions such as heavy rain or snowfall, which can result in turbulent and/or low visibility conditions for the MHA. Additionally, as underserved regions further develop reliable transportation and utility infrastructure, thus making the MHA less essential in that particular region, such a threat can be mitigated by shifting activity to other regions that lack such infrastructure and would benefit from the MHA services [4]. Also, societal norms, cultural expectation, low literacy rates, and limited public education need to be addressed to ensure sustainability of the program. Finally, economic sustainability can be a threat to any public health program, particularly if there is a dependency on donors and public funds. Efforts need to be made on creating an economic model that sustains services through consistent revenues and concrete deliverables to the public.

## FUTURE DIRECTIONS

The airship’s high impact potential, infrastructure, and innovation of the multi-clinic design contribute to the sustainability of the MHA program, however, involvement of both local and international stakeholders is fundamental to promote sustainability. Therefore, future recommendations for the MHA program include establishing a wide representation from diverse internal and external stakeholders, developing a two-way relationship with existing local health care facilities, and strengthening this relationship by creating opportunities to involve local community members and health care workers.
